# TriTECM: A tetrafunctional T-cell engaging antibody with built-in risk mitigation of cytokine release syndrome

**DOI:** 10.3389/fimmu.2022.1051875

**Published:** 2022-11-10

**Authors:** Stefania C. Carrara, Julia Harwardt, Julius Grzeschik, Björn Hock, Harald Kolmar

**Affiliations:** ^1^ Institute for Organic Chemistry and Biochemistry, Technische Universität Darmstadt, Darmstadt, Germany; ^2^ Ferring Darmstadt Laboratory, Biologics Technology and Development, Darmstadt, Germany; ^3^ Ferring Biologics Innovation Centre, Epalinges, Switzerland; ^4^ Aerium Therapeutics, Epalinges, Switzerland; ^5^ Centre for Synthetic Biology, Technische Universität Darmstadt, Darmstadt, Germany

**Keywords:** T-cell engager, multispecific antibody, cytokine release syndrome, IL-6R, CRS, tetraspecifics

## Abstract

Harnessing the innate power of T cells for therapeutic benefit has seen many shortcomings due to cytotoxicity in the past, but still remains a very attractive mechanism of action for immune-modulating biotherapeutics. With the intent of expanding the therapeutic window for T-cell targeting biotherapeutics, we present an attenuated trispecific T-cell engager (TCE) combined with an anti- interleukin 6 receptor (IL-6R) binding moiety in order to modulate cytokine activity (TriTECM). Overshooting cytokine release, culminating in cytokine release syndrome (CRS), is one of the severest adverse effects observed with T-cell immunotherapies, where the IL-6/IL-6R axis is known to play a pivotal role. By targeting two tumour-associated antigens, epidermal growth factor receptor (EGFR) and programmed death ligand 1 (PD-L1), simultaneously with a bispecific two-in-one antibody, high tumour selectivity together with checkpoint inhibition was achieved. We generated tetrafunctional molecules that contained additional CD3- and IL-6R-binding modules. Ligand competition for both PD-L1 and IL-6R as well as inhibition of both EGF- and IL-6-mediated signalling pathways was observed. Furthermore, TriTECM molecules were able to activate T cells and trigger T-cell-mediated cytotoxicity through CD3-binding in an attenuated fashion. A decrease in pro-inflammatory cytokine interferon γ (IFNγ) after T-cell activation was observed for the TriTECM molecules compared to their respective controls lacking IL-6R binding, hinting at a successful attenuation and potential modulation *via* IL-6R. As IL-6 is a key player in cytokine release syndrome as well as being implicated in enhancing tumour progression, such molecule designs could reduce side effects and cytotoxicity observed with previous TCEs and widen their therapeutic windows.

## Introduction

Therapeutic strategies for solid cancers have led to the rise of monoclonal antibodies (mAbs), targeting a single target that is overexpressed on tumour cells. Moreover, bispecific antibodies (BsAbs) and multispecific antibodies are now gaining importance due to their power of binding multiple tumour-associated antigens (TAA) simultaneously and/or redirecting immune cells to the tumour microenvironment to enhance anti-tumour activities and combining different mechanisms of action (1–4). Besides other immune cells, T lymphocytes (T cells) have been a central focus point in the battle against cancer in recent years. A plethora of T cell-based immunotherapies showing moderate to good success, particularly in hematologic cancers, have been developed and approved, including the renowned immune-checkpoint inhibitors and engineered chimeric antigen receptor T cells (CAR-Ts) (5, 6).

A special class of BsAbs termed bispecific T-cell engagers (BiTEs) was introduced by Micromet (now owned by Amgen Inc.) in 2008, where T cells are redirected to tumour cells through the binding of both a TAA and a T cell surface antigen, allowing for enhanced tumour lysis and redirecting of T cells to the tumour microenvironment (7–9). BiTEs consist of two linked single-chain variable fragments (scFvs) and are thus relatively small molecules with a short half-life. The first-in-class BiTE molecule to be approved for therapy was Blinatumomab (Blincyto) in 2014 for the treatment of B-cell malignancies by simultaneously targeting CD19 on B cells and CD3 on T-cells (7, 10–13). Another T cell-engaging bispecific molecule approved for clinical use in the European Union is catumaxomab (Removab) of the TrioMab format, which targets epithelial cell adhesion molecule (EpCAM) and CD3 with a functional Fc region mediating effector functions resulting in a trifunctional antibody (14, 15). While the breakthrough of bispecific T-cell engagers was validated with the regulatory approval of both molecules, they both have shortcomings. Catumaxomab, a rat-mouse hybrid IgG2 antibody with a very high affinity for CD3, led to T cell over-activation and cytokine release syndrome (CRS) and was later voluntarily withdrawn due to commercial reasons (16). Similarly, treatment with blinatumomab was reported to be associated with a high risk of CRS, narrowing its therapeutic window and additionally requiring frequent infusion due to its short half-life (17–21). The first monoclonal antibody ever approved for clinical use, muromonab-CD3 (Orthoclone-OKT3), a mouse antibody binding CD3, was deployed for the treatment of acute kidney allograft rejection and additionally investigated for its use against T cell acute lymphoblastic leukemia (22). In 2010, the manufacturing of Muromonab-CD3 was voluntarily withdrawn due to severe side effects after administration and the growing number of better-tolerated alternatives that were available (23). It was also the coiner for the term “cytokine release syndrome” back in the 1990s (19, 24, 25).

Cytokine release syndrome is one of the most frequent grave adverse effects of T cell-engaging immunotherapies, resulting in a systemic inflammatory response after immunotherapy (19). Though grade 1 and 2 CRS result in mild reactions such as fever and hypotension, intravenous fluids or low-dose vasopressors are generally required. Grade 3 CRS results in hospitalisation, high-dose vasopressors are administered, and signs of organ dysfunction appear to begin. Lastly, severe CRS, namely grade 4, embodies life-threatening situations where mechanical ventilation support is required, grade 4 organ toxicities are observed, and occurring hypotension requires the application of multiple high-dose vasopressors (19, 26–28). While the pathophysiology of CRS is not wholly understood, interleukin-6 (IL-6), interleukin-10 (IL-10), and interferon-γ (IFNγ) are known to be the main drivers of CRS. Initially, activated T cells release IFNγ which is secreted and in turn activates macrophages, leading to the excessive production of IL-6, IL-10, and TNFα (19, 29). While all cytokines presumably play an important role, IL-6 seems to be a key player in the pathophysiology of CRS and contributes to many key symptoms (19, 30, 31). Currently, grade 3 or 4 CRS are managed by treating patients with tocilizumab, a therapeutic anti-IL-6R antibody that blocks receptor activation by IL-6. Tocilizumab (Actemra) was approved by the U.S. Food and Drug Administration (FDA) in 2010 for rheumatoid arthritis treatment and more recently received emergency use authorisation for the management of CRS (32–34). Furthermore, in light of the unprecedented COVID-19 pandemic where life-threatening infections were observed resulting in CRS, not only tocilizumab but also sarilumab, another antibody directed against IL-6R, have gained importance in the management of CRS in critically ill patients (35–42). Although risk- and grade-management of CRS with the administration of tocilizumab appears to work well, improvements are required to widen the therapeutic index of immunotherapies and boost their efficacies, particularly in solid tumours.

Due to IL-6’s pleiotropic nature, it has also been shown to play critical roles in tumour growth, angiogenesis, and metastasis of different cancer types by activating signalling pathways after assembly and dimerization with its receptor IL-6R and glycoprotein 130 (gp130) (43). The IL-6/IL-6R/gp130 complex is able to activate signal transduction by the Janus kinase/signal transducer and activator of transcription (JAK/STAT) pathway by either membrane-bound IL-6R and gp130 (classical signalling) or by soluble forms of IL-6R (sIL-6R) that then joins membrane-bound gp130 (*trans* signalling) to mitigate downstream signalling (44). The JAK/STAT pathway plays a critical role in solid tumour progression and thus inhibition of downstream events by IL-6 or IL-6R blockage is a promising strategy for the development of new anti-cancer combination therapies (43, 45). Furthermore, co-targeting of the EGFR and IL-6R pathways through blockage of oncogenic signalling may aid in overcoming acquired EGFR resistance that is observed after treatment with anti-EGFR drugs such as small molecule inhibitors gefitinib or erlotinib (46–49).

Tumour-associated macrophages (TAMs) are a key element of the heterogenous tumour microenvironment (TME) and can take on both tumour-impairing or tumour-promoting roles by differentiation of infiltrating or resident macrophages to either M1- or M2-type TAMs, respectively (50). Previous studies have shown the implication of TAMs and their ability to produce IL-6 to promote tumorigenesis in hepatocellular carcinoma stem cells (51), K-ras mutant lung cancer mouse models (52), and breast cancer (44). Under hypoxic conditions in an immunosuppressive TME, upregulation of IL-6R expression on tumour cells and increased IL-6 production have been reported, inducing M2-TAMs and further promoting tumour progression and expansion, and survival resistance (51, 53, 54). The complexity of the TME culminates in the difficulty of efficaciously targeting tumours by monospecific therapies due to the involvement of many key players. Thus, combinatorial approaches through multispecific antibodies targeting multiple pathways might encourage redefining and reprogramming the TME to efficaciously eradicate tumours, and inhibiting TAM-derived IL-6-mediated signalling transduction pathways. Eventually, this strategy may contribute to the activation of immunologically cold tumours (55).

While many antibody formats exist for T cell-redirecting antibodies, the preclinical and clinical efficacy of such antibodies is often hampered by high toxicities in early stages. In recent years, researchers have investigated the role of CD3 affinity to improve the therapeutic index of T-cell engagers (TCEs) (56–60). This requires the development and characterisation of novel CD3 binders with less critical cytokine release profiles. Trispecific T-cell engager (TriTE) antibodies have been described in literature as next-generation T-cell engager therapies, though studies in humans are yet to be concluded (7, 61, 62). For example, in the antibody described by Tapia-Galisteo and colleagues, a CD3-specific single-chain variable fragment (scFv) was flanked by two different tumour-targeting V_HH_ antibody fragments directed against epidermal growth factor receptor (EGFR) and EpCAM for the treatment of colorectal cancer (61). Another novel trispecific T-cell engager concept was described by Wu and co-workers by binding not only to CD3 but also its co-stimulatory receptor CD28 by using the cross-over dual variable (CODV) bispecific antibody format and additionally targeting CD38 on myeloma cells (62). Moreover, eight TCE mAbs are in late-stage clinical studies targeting several different TAA for different indications, including CD7, BCMA, CD20, and CD123 (63). A further three candidates are currently undergoing regulatory review, namely teclistamab (BCMAxCD3), glofitamab (CD20xCD3), and teplizumab (humanised OKT3) (64). Two additional bispecific molecules were approved for therapeutic use in the USA and the EU in 2022, targeting gp100xCD3 as a bispecific fusion protein (tebentafusp), and CD20xCD3 as a bispecific antibody (mosunetuzumab). The vast number of preclinical and clinical programs investigating CD3-binding either as mono- or bispecific antibodies further highlights the unmet need and interest in pursuing T cell-activating immunotherapies.

To overcome the limitations of current clinical approaches, we present a first-in-class trispecific T-cell engager and TME and cytokine modulator (TriTECM), by engineering a two-in-one dual-TAA targeting antibody with anti-CD3 and anti-IL-6R binding moieties. This tetrafunctional antibody results in high tumour selectivity by targeting EGFR and the checkpoint inhibitor programmed death-ligand 1 (PD-L1) simultaneously, and has attenuated CD3ϵ binding, diminishing the cytokine release and toxicity effects. On top, an anti-IL-6R binding moiety that modulates the signalling of IL-6 released after T cell and macrophage activation is present to not only potentially support tumour eradication but also to mitigate CRS events. This concept may allow the usage of previously developed high potency anti-CD3 antibodies in the long term, obviating the need to discover novel functionally attenuated CD3-binders. For the first time, to the best of our knowledge, an anti-IL-6R moiety is fused directly to a trispecific T cell-engager to inhibit both *cis*- and *trans-*IL-6-mediated signalling, aimed at directly modulating but not completely abolishing cytokine release after T-cell activation.

## Materials & methods

### Cell culture

A431 (ACC 91), A549 (ACC 107), and THP-1 (ACC 16) cells were obtained from DSMZ (German Collection of Microorganisms and Cell Cultures GmbH). Adherent cell lines (A431, A549) were maintained in Dulbecco’s Minimal Eagle Medium (DMEM) supplemented with 10% FBS and 1x penicillin/streptomycin. THP-1 cells were maintained in RPMI 1640 supplemented with 10% FBS and 1x penicillin/streptomycin. Cells were subcultured every 2-3 days and incubated at 37°C, 5% CO_2_.

### Cloning, production and purification of antibody constructs

The HCP-LCE IgG backbone was used as a starting block (65). For the different scFvs, gene fragments were ordered from Twist Biosciences based on their published protein sequences (66) with respective *Sap*I overhangs. The various architectures were cloned using Q5 DNA Polymerase and subsequent *Sap*I-mediated Golden Gate Cloning (GGC). The sequences were verified by Sanger Sequencing at Microsynth (Göttingen). An effector-silenced Fc-backbone carrying LALA mutations was used (67), as well as the Knob-into-Hole (KiH) technology to ensure heterodimerisation (68).

For the production of the antibodies, Expi293-F (Thermo Fisher Scientific) cells were transfected with Expifectamine293 (Thermo Fisher Scientific), following the manufacturer’s instructions. The cells were cultured in Expi293 Expression Medium (Thermo Fisher Scientific), sub-cultured every 3-4 days and incubated at 37°C, 8% CO_2_. Transfections were harvested 5 days post-transfection. The supernatants were sterile filtered and supplemented with 18.1 mL/L BioLock (IBA Lifesciences). Purification of all KiH variants was performed by a two-step purification using an ÄKTA Pure25 (Cytiva Lifesciences); a His-tag purification using a HisTrap™ excel column (Cytiva Lifesciences) was performed followed by TwinStrep^®^-tag purification using a Streptactin^®^XT 4Flow^®^ column. In this manner, heterodimeric heavy chains were ensured.

### Biophysical characterisation (SDS-PAGE, Thermal stability)

To characterise the produced antibodies, SDS-PAGE analysis was performed. To this end, 4 µg purified mAb were loaded onto a Mini-PROTEAN TGX 4-15% Gel (BioRad) with either reducing- or non-reducing-Lämmli buffer, and subsequently stained with Coomassie.

For thermal stability determination, antibodies were incubated with SYPRO Orange (Thermo Fisher Scientific) and a thermal shift assay was performed using a CFX Connect Real-Time PCR System (BioRad). The temperature gradient was set from 10°C to 95°C with an increment of 0.5°C/10 s. The derivatives of the melt curves were determined with the CFX Maestro software to determine the melt temperature (T_M_).

### Affinity determination, simultaneous binding and competition assays *via* BLI

Affinities were determined using biolayer interferometry (BLI) on an Octet RED96. For kinetics determination, 60 nM antibody was loaded onto anti-human Fc capture (AHC) biosensors and incubated with varying concentrations of antigen, in a range from 0 – 500 nM for EGFR and PD-L1, and 0 – 200 nM for IL-6R. Fitting of the curves for affinity determination was performed based on Savitzky-Golay filtering and a 1:1 Langmuir binding model.

For simultaneous binding studies with BLI, 10 µg/ml antibody was loaded onto an AHC biosensor and sequentially associated to 200 nM IL-6R, followed by 500 nM EGFR and PD-L1. A negative control with PBS was included to eliminate unspecific binding of the self-produced antigens.

Competition assays for PD-L1/PD-1 and IL-6/IL-6R were performed as previously described (65, 69, 70).

### T-cell activation assays

To determine the potency of T-cell engagement, the T-cell activation Bioassay (NFAT) (Promega) was performed following the manufacturer’s instructions. In brief, 4 x 10^4^ A431 cells/well were seeded in sterile 96-well plates and allowed to adhere overnight. The following morning, 1 x 10^5^ effector cells/well were added with the indicated antibody dilutions (3x concentrations). The co-culture was incubated for 6 h at 37°C, 5% CO_2_, followed by the addition of Bio-Glo reagent. After 5-10 min, the plate was measured on a luminescence plate reader.

### PBMC isolation

Peripheral blood mononuclear cells (PBMCs) were obtained from buffy coats from healthy human donors obtained from the Deutsche Rotes Kreuz (Frankfurt). To this end, 25 ml blood was mixed 1:1 with PBS- 2% FBS (PBS-F) and PBMCs were purified using SepMate-50 tubes following the manufacturer’s instructions (StemCell Technologies). The isolated PBMCs were frozen in 70% RPMI 1640, 20% FBS and 10% DMSO and thawed directly when required. All work was performed according to local ethics and welfare regulations.

### Cytokine release assay

For cytokine release assays, PBMCs were thawed and seeded onto 48-well plates at 3 x 10^5^ cells/well. The desired antibody concentration was added to the cells, and the mixture was incubated at 37°C, 5% CO_2_ for 72 h. After 72 h, the supernatant was collected and centrifuged to remove any cell debris. The cells were separated for further flow cytometric analysis. A minimum of four healthy donors were used and at least biological duplicates were measured in independent experiments.

### Signalling assays

To detect phosphoproteins and signalling inhibition, HTRF (Perkin Elmer) kits were used. Therefore, 5 x 10^4^ A549 cells/well were seeded onto 96-well tissue-culture plates and allowed to adhere for 4 h, cells were subsequently serum-starved overnight. The serum-starved cells were pre-treated with the desired antibody concentrations for 1 h before the addition of 20 ng/ml ligand for 10 or 40 min for EGF and IL-6, respectively. Immediately afterwards, the cells were washed with ice-cold PBS and lysed in the appropriate lysis buffer according to the manufacturer’s instructions. The investigation of the EGF/EGFR pathway was performed using the Phospho-Akt (Ser4739 cellular HTRF Kit (Perkin Elmer), and the IL-6/IL-6R pathway was investigated using the Phospho-STAT3 (Tyr705) cellular HTRF kit (Perkin Elmer). Data was graphed using GraphPad Prism 8.0.1. Assays were repeated at least three times with biological duplicates.

### Cytokine ELISAs

Quantification of secreted cytokines from cell culture supernatants was measured using commercially available ELISAs following the manufacturer’s instructions. ELISA kit was purchased from R&D Systems: Human IFN-gamma DuoSet ELISA (DY285B).

### T-cell-mediated cytotoxicity

Evaluation of cytotoxicity was performed by incubating A431 cells with PBMCs (effector-to-target ratio 10:1) in 96-well tissue culture plates with the desired antibody concentration for 24 – 48 h. After different time points, the supernatant was used to measure released LDH by the LDH-Glo Cytotoxicity Assay (Promega) following the manufacturer’s instructions. Plates were observed under a bright-field microscope to evaluate potential cell killing. Further, dead cell staining was performed by trypsinising and collecting all cells, staining with propidium iodide (PI) (Sigma Aldrich) and measuring PE fluorescence using a flow cytometer.

### Flow cytometry

Flow cytometric analysis was performed by first washing the cells of interest with ice-cold PBS- 2% FBS (PBS-F). Cells were then stained with primary antibodies and incubated on ice for 30 min. Subsequently, the cell pellets were washed with ice-cold PBS-F and resuspended in an appropriate volume of PBS-F for measurement using a CytoFlex S (Beckman Coulter) flow cytometer. If the primary antibody was not directly conjugated, a secondary antibody incubation was performed on ice for 15 min before measurement. The antibodies used in this study were: anti-human Fc-PE (Thermo Fisher Scientific), anti-human CD69-FITC (Thermo Fisher Scientific), anti-human CD25-APC (Thermo Fisher Scientific). Dot plots were generated using FlowJo V10 software.

## Results

### Design & generation of TriTECM variants

In this proof-of-concept study, we combined existing and well-characterised binding modules in different antibody formats to generate a trispecific T-cell engager with TME and cytokine release modulation (TriTECM). Due to its ability to bind and block both EGFR and PD-L1 pathways and having two binding sites for each target, a recently isolated two-in-one antibody (65) was chosen as the backbone, carrying an effector-silenced Fc to minimise cytotoxicity and extend the half-life. This molecule carries dual targeting abilities in a generic IgG1 antibody format, where the light chain mainly contributes to EGFR-binding and the heavy chain to PD-L1-binding. Moreover, while individual binding to each target occurs with moderate affinity, tumour cells expressing both antigens were bound with high affinity, indicating that the antibody, besides being able to inhibit the PD-1/PD-L1 axis, displays enhanced tumour selectivity.

For CD3ϵ-engagement, the fully human monoclonal antibody foralumab (NI0401, abbreviated as αCD3ϵ) was considered (71, 72), from which a single-chain variable fragment (scFv) was derived. Clinical investigations have been performed using different formulations of foralumab for several indications, including non-alcoholic steatohepatitis (NASH), type 2 diabetes mellitus, primary and secondary multiple sclerosis, and more recently for COVID-19 infections (71, 73). According to a reported study by Moreira et al., nasal administration of foralumab in a pilot study using patients with mild to moderate COVID-19 disease severity led to no CRS and lowered IL-6 blood levels 10 days after administration (72). For this proof-of-concept study, the anti-CD3 scFv was used as a CD3-based T-cell engager.

For inhibition of the IL6-IL-6R-gp130 pathway, a sarilumab-derived scFv (abbreviated with αIL-6R) was generated as sarilumab exhibits exceptional affinity to the IL-6Rα with 61.9 pM, approximately 20-fold higher than tocilizumab, and is able to block both the classical- and trans-mediated signalling pathways (74). Furthermore, recent studies showed its ability to modulate CRS events in SARS-CoV2-infected patients (35–42).

**Figure 1:**
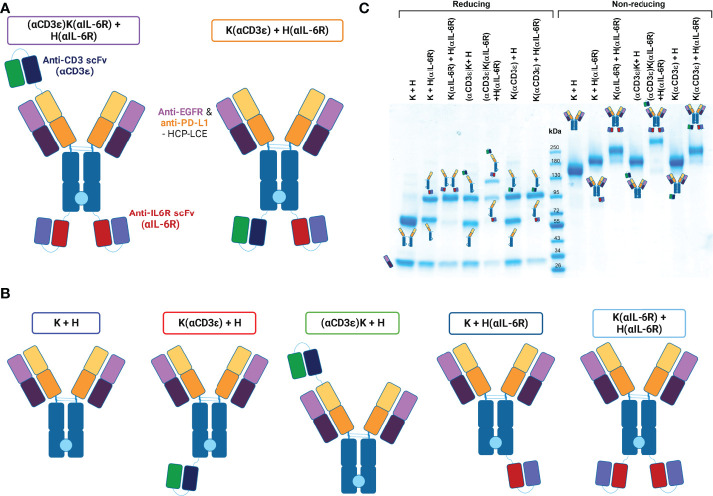
Conceptual architecture of TriTECMs. **(A)** Molecular design of two TriTECM constructs, (αCD3ϵ)K(αIL-6R) + H(αIL-6R) as scFv(VH)-IgG1(H)-scFv^2^ (purple) and K(αCD3ϵ) + H(αIL-6R) as an IgG1(H)-scFv^2^ (orange). The blue/green variable regions represent the anti-CD3 NI0401-derived scFv, the red/blue variable regions represent the anti-IL-6R sarilumab-derived scFv (αIL-6R), the Fabs originate from HCP-LCE (65) and an effector-silenced IgG1 backbone was used. The light blue circle between the CH3 domains represent the Knob-into-Hole technology for heterodimerisation. **(B)** Control variants with the same colour-coding of the Fab and scFv regions as in a). The boxes represent the colours used to represent them hereafter. **(C)** SDS-PAGE analysis under reducing (left) and non-reducing (right) conditions for all variants. The schematic representation of either the reduced heavy and light chains or the entire molecule are depicted.

With these building blocks, different TCEs were designed. To have better control over the positioning of the scFvs added, all molecules were designed with the Knob into Hole (KiH) technology (68). To avoid hyper-cytokine release, a single CD3-binding scFv was fused onto the HCP-LCE KiH backbone. As well recognised, the distance and relative positioning of TAA and CD3 binding modules of T-cell recruiting antibodies matter, influencing their ability to bridge the immune synapse between a tumour-associated antigen and the TCR on the surface of T cells (56, 75). For this purpose, a classical approach was taken, fusing the anti-CD3ϵ scFv to the N-terminus of the VH domain with a (G_4_S)_3_ linker, providing a relatively small distance between CD3-engagement and the surface of the tumour cell upon binding. Conversely, a C-terminal fusion to the Knob-Fc with a (G_4_S)_3_ linker was also generated, providing the largest distance between the tumour-binding Fab and the engaging T cell (**Figure 1A**). The anti-IL-6R scFv was placed either at the C-termini of both heavy chain (HC) Fc-fragments or only at the Hole-Fc heavy chain, respectively. The control variants are all depicted and named in **Figure 1B**. For simplicity, the nomenclature is based on the positioning of the scFvs (either N- or C-terminal) to the heavy chain and either for the Knob (K) or Hole (H) HCs. As an example, a fusion of anti-CD3ϵ scFv N-terminal to the VH of the Knob HC together with a C-terminal fusion of the anti-IL-6R scFv to both the Knob and Hole HC is denoted as (αCD3ϵ)**K**(αIL-6R) + **H**(αIL-6R). The parental two-in-one antibody is termed K + H.

### Biophysical characterisation of variants

Production of the antibodies was performed in Expi293-F cells by transient transfection with expression plasmids using Expifectamine293. To ensure exclusive isolation of heterodimers, a Twin-StrepII-tag sequence was placed at the C-terminus of the CH3 domain of the Knob-Fc, while a His_6_-tag was placed C-terminally to the Hole-Fc, as previously described (76). Subsequently, the cell culture supernatant was purified *via* a two-step purification, namely an IMAC followed by StrepTactin purification to purify only heterodimeric antibodies carrying both a Knob- and a Hole-HC. SDS-PAGE analysis revealed all the expected heavy- and light-chain bands under reducing conditions, as well as the expected molecular size under non-reducing conditions with no degradation products (**Figure 1C**). The antibodies unveiled appropriate yields for all variants, with the yield decreasing with increasing number of fused scFvs as expected (**Table 1**). Thermal stability investigated by SYPRO Orange revealed melting temperatures between 63.50°C and 78.00°C, with the parental K + H exhibiting 66.50°C, revealing no large reduction in thermal stability (**Table 1**; **Supplemetary Figure 1**).

**Table 1:** Yields and thermal stability of all variants.

Variants	Yield per 1 L (mg)	T_M_ (°C)
**K + H**	122.344	66.50
**K + H(αIL-6R)**	54.512	70.00
**K(αCD3ϵ) + H**	63.170	78.00
**K(αCD3ϵ) + H(αIL-6R)**	22.478	69.50
**K(αIL-6R) + H(αIL-6R)**	31.635	63.50
**(αCD3ϵ)K + H**	101.765	74.50
**(αCD3ϵ)K(αIL-6R) + H(αIL-6R)**	23.643	64.00
**oa(αCD3ϵ)K(αIL-6R)**	26.074	71.00

Affinity towards the different antigens was determined *via* biolayer interferometry (BLI). Antibodies were loaded onto AHC biosensors and varying concentrations of antigen were associated to the mAb. The determined affinities were in similar ranges to those cited in literature from previous studies (65, 74), with only minor changes where the fused scFvs could presumably interfere with binding (**Table 2**; **Supplementary Figure 2**). Nevertheless, the affinity to neither EGFR nor PD-L1 was greatly influenced by N- or C-terminal scFv fusions. The affinity towards soluble IL-6R (sIL-6R-TS) was not determined for all IL-6R-binding variants due to the very slow dissociation (**Supplementary Figure 2**), however both K(αCD3ϵ) + H(αIL-6R) and (αCD3ϵ)K(αIL-6R) + H(αIL-6R) showed single-digit nanomolar affinity towards IL-6R, substantiating the very high affinity of the full-length variant from literature (74).

**Table 2:** Affinities towards soluble, monomeric EGFR, PD-L1 or IL-6R as determined by BLI.

Variant	K_D_ (nM)		
	His_6_-EGFR-TS	His_6_-PD-L1-TS	sIL-6R-TS
**K + H**	173 ± 10.8	92.9 ± 2.35	–
**K + H(αIL-6R)**	355 ± 19.4	68.8 ± 2.02	< 0.001*
**K(αIL-6R) + H(αIL-6R)**	503 ± 20.0	70.7 ± 2.51	< 0.001*
**K(αCD3ϵ) + H**	228 ± 12.4	105.0 ± 2.80	–
**K(αCD3ϵ) + H(αIL-6R)**	172 ± 10.5	112 ± 3.14	1.80 ± 0.188
**(αCD3ϵ)K + H**	236 ± 11.5	108.0 ± 2.85	–
**(αCD3ϵ)K(αIL-6R) + H(αIL-6R)**	271 ± 14.4	130 ± 3.73	2.71 ± 0.0848

*curves were not fittable due to very slow dissociation that precludes exact off-rate determination. TS – TwinStrepII tag.

### On-cell affinities and simultaneous binding

To ensure the tumour-targeting ability of the two-in-one backbone remained intact, A431 cells were examined that are both positive for EGFR and PD-L1, revealing very similar on-cell affinities for all variants (**Supplementary Table 1**; **Supplementary Figure 3**). The parental K + H antibody showed an affinity of 2.5 nM, similar to previous studies (65), and the two TriTECM molecules revealed 12.3 and 21.2 nM for (αCD3ϵ)K(αIL-6R) + H(αIL-6R) and K(αCD3ϵ) + H(αIL-6R), respectively. Nonetheless, very good on-cell affinities were measured for all variants. CD3 binding was observed on Jurkat cells, with the (αCD3ϵ)K + H and K(αCD3ϵ) + H variants binding with two-digit nanomolar affinities. A reduction in CD3-binding was observed for both TriTECM variants, presumably due to different conformations of the molecules (**Supplementary Table 1**; **Supplementary Figure 3**). Lastly, IL-6R-binding was confirmed on macrophage-like THP-1 cells (**Supplementary Table 1**; **Supplementary Figure 3**).

**Figure 2:**
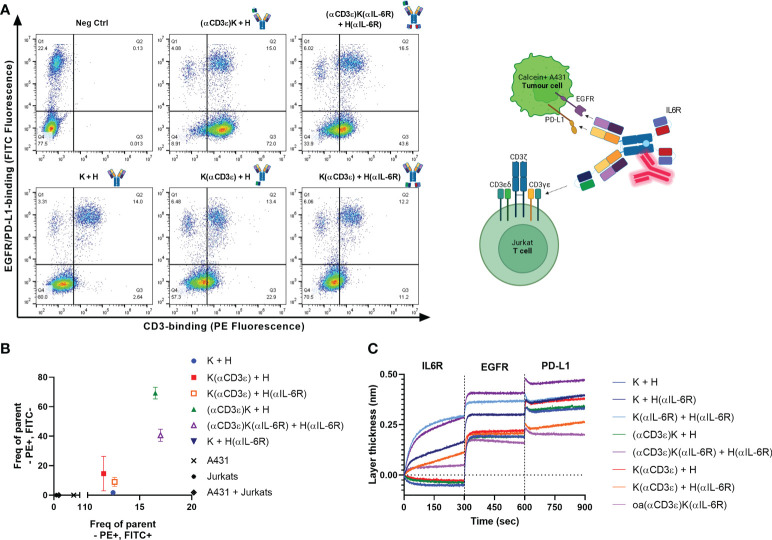
Simultaneous binding to all targets. **(A)** Dot plots for simultaneous binding of Calcein AM-stained A431 cells (y-axis) and bound Jurkat cells (x-axis). Binding to Jurkats *via* total CD3 was detected using anti-human Fc PE. Events in Q1 represent the Calcein AM-stained A431 cells (FITC^+^/PE^-^), while those in Q4 represent Jurkats without CD3-binding (FITC^-^/PE^-^). A shift towards Q2 (FITC^+^/PE^+^) depicts binding of the A431 cells after incubation with Jurkats, and Q3 shows CD3-binding on Jurkats (FITC^-^/PE^+^). 20’000 events are displayed per dot plot. **(B)** Quantification of events in Q3 (FITC^-^/PE^+^) versus Q2 (FITC^+^/PE^+^) from **(A)** Positive events in both quadrants ensures Jurkat & A431 engagement. **(C)** Investigation of simultaneous binding *via* BLI. Antibodies were loaded onto AHC biosensors and 200 nM IL-6R, followed by 500 nM of EGFR and PD-L1 were associated sequentially for 300 seconds each.

For T-cell engagers, simultaneous binding and engagement of both tumour- and T-cells is crucial. Accordingly, simultaneous binding of Calcein AM-stained A431 tumour cells and pre-incubated CD3-stained Jurkat cells was investigated by flow cytometry (**Figure 2A**). Quantification of FITC^+^/PE^+^ and FITC^-^/PE^+^ binding events were considered as successful co-binding, as both CD3-binding on Jurkat cells was observed through the antibodies, as well as to A431 cells *via* EGFR and PD-L1 (**Figure 2B**). The two best candidates were (αCD3ϵ)K + H and (αCD3ϵ)K(αIL-6R) + H(αIL-6R), followed by K(αCD3ϵ) + H and K(αCD3ϵ) + H(αIL-6R). Weaker simultaneous binding was observed for (αCD3ϵ)K(αIL-6R) + H(αIL-6R) and K(αCD3ϵ) + H(αIL-6R) compared to their counterparts without the anti-IL-6R scFv, as expected from their reduced on-cell affinity, however still significant compared to K + H or K + H(αIL-6R) which are not able to bind Jurkat cells due to the absence of the CD3 binding module.

Furthermore, simultaneous binding to IL-6R, EGFR and PD-L1 was confirmed *via* BLI by loading the different constructs onto AHC biosensors and associating to the three antigens sequentially for 300 seconds each (**Figure 2C**). A one-armed variant of (αCD3ϵ)K(αIL-6R) + H(αIL-6R) (dubbed oa(αCD3ϵ)K(αIL-6R)) was generated to test whether a single arm could still bind all three targets. Interestingly, the one-armed variant was also able to bind IL-6R, EGFR and PD-L1, though with decreased layer thickness increase due to only monovalent binding to each antigen. This indicated that the two-in-one Fab retained its ability to simultaneously bind EGFR and PD-L1 with scFvs fused to its N-terminus. Overall, the tetraspecificity of both TriTECM molecules was confirmed.

### Ligand competition and signalling pathway inhibition

**Figure 3:**
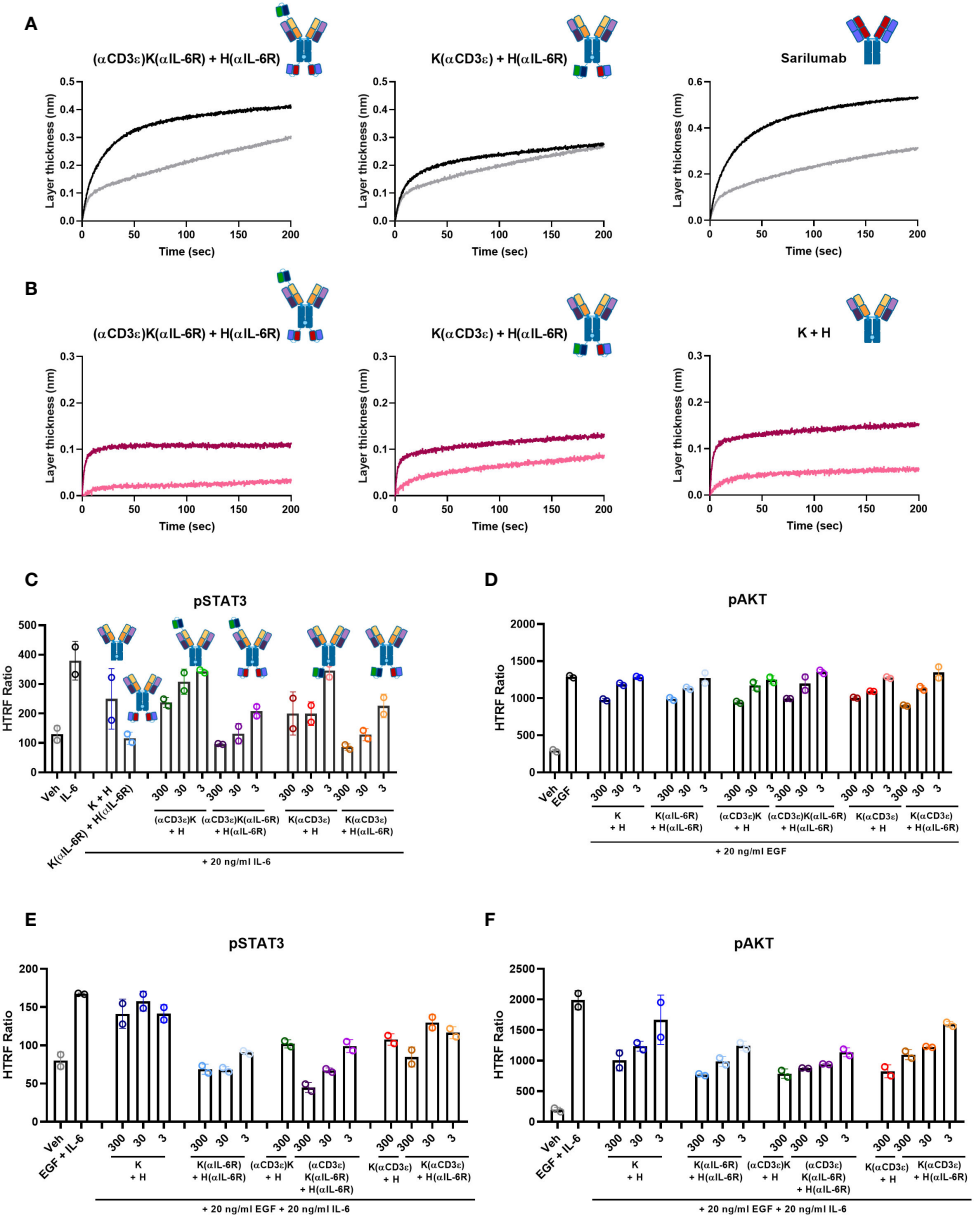
Ligand competition and signalling inhibition. **(A)** IL-6/IL-6R competition assay by BLI. B) PD-L1/PD-1 competition assay by BLI. **(A, B)** The antibodies were loaded onto FAB2G biosensors and associated either to the receptor alone (black or dark pink) or pre-incubated with ligand (grey or light pink). **(C–F)** HTRF assays for signalling activation. A549 cells were pre-incubated with the indicated amounts of antibodies and then stimulated with the respective ligands. pSTAT3 was measured for IL-6-mediated signalling **(C)** or combination of EGF- and IL-6 stimulation **(E)**. pAKT was measured for EGF-mediated signalling **(D)** or combination of EGF- and IL-6 stimulation **(F)**. The HTRF ratio is presented, which is calculated as $\frac{Emission\left(665\;nm\right)}{Emission\;\left(620\;nm\right)}\times 10000$ . Biological duplicates were measured, with the individual data points representing individual measurements and error bars representing the SD of biological duplicates. Graphs were plotted using GraphPad Prism 8.0.1.

For a full therapeutic effect, it is important that the antibodies binding to EGFR, PD-L1 and IL-6R have antagonistic effects. Sarilumab is well-known to compete with the IL-6 binding site on the IL-6R (77), and the previously identified parental K + H antibody competes with PD-1 for binding to PD-L1 (65). *Via* BLI, a competition assay was performed for both TriTECM molecules and full-length sarilumab or K + H as controls. For IL-6/IL-6R competition, the (αCD3ϵ)K(αIL-6R) + H(αIL-6R) construct inhibited equally well compared with sarilumab IL-6R binding and ligand competition, however, the K(αCD3ϵ) + H(αIL-6R) showed weaker competition (**Figure 3A**). This is presumably due to monovalent IL-6R binding, whereas both sarilumab and (αCD3ϵ)K(αIL-6R) + H(αIL-6R) are bivalent. For PD-L1, all antibodies displayed good binding to PD-L1, which became weaker upon pre-incubation with PD-1, confirming competition of binding (**Figure 3B**).

As the parental K + H antibody binds to domain II of EGFR, it does not compete with its ligand EGF. However, it still inhibits the EGF-mediated signalling pathway (65). Similarly, sarilumab is able to inhibit IL-6-mediated signalling (77). Hence, pAKT and pSTAT3 were measured for EGF- and IL-6-mediated signalling inhibition, respectively. For IL-6/IL-6R, pSTAT3 analysis by HTRF assays revealed dose-dependent inhibition for both TriTECMs and the K(αIL-6R) + H(αIL-6R) control mAb (**Figure 3C**). While a slight reduction was noticed with 300 nM of either (αCD3ϵ)K + H or K(αCD3ϵ) + H, the inhibition was not as high as for the molecules with the anti-IL-6R scFv and similar to that of K + H. For EGF/EGFR signalling, pAKT was measured and all antibodies exhibited dose-dependent inhibition compared to the EGF control, all very similar to the parental K + H antibody (**Figure 3D**). Furthermore, to investigate whether the antibodies could inhibit both pathways simultaneously, cells were pre-incubated with mAbs and then stimulated with a combination of EGF and IL-6. HTRF analysis revealed dose-dependent inhibition of both pathways as seen by pSTAT3 and pAKT inhibition (**Figures 3E, F**). Thus, despite minor differences in EGFR/PD-L1 binding of the TriTECMs as measured by affinity determination or on-cell affinities, their functionalities were retained compared to the parental two-in-one antibody in the K + H format.

### T-cell activation

**Figure 4:**
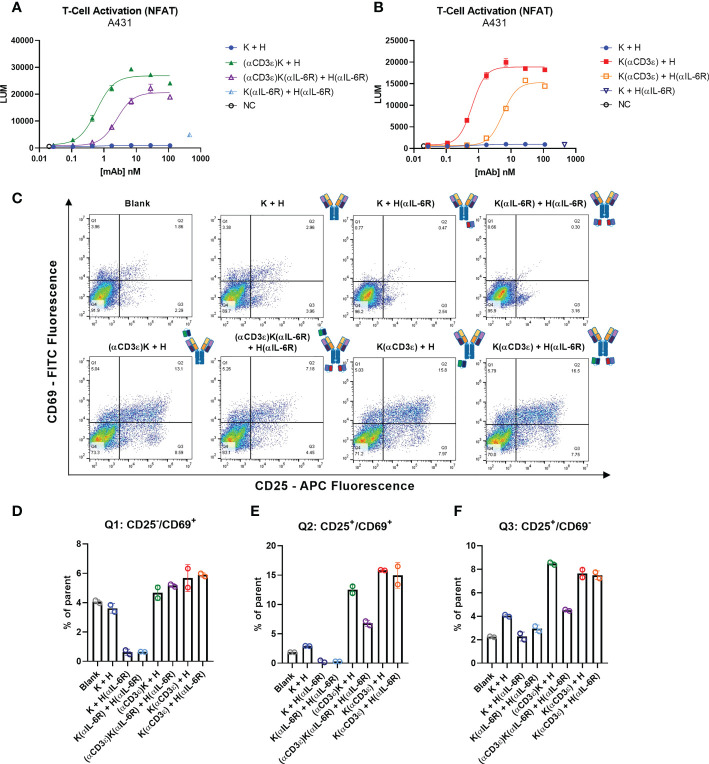
T-cell activation. **(A, B)** T-cell activation bioassay NFAT pathway with A431 as target cells. **(C)** Dot plots for measurement of CD25-APC (x-axis) and CD69-FITC (y-axis) of total CD3^+^ T cells from PBMCs after 48 h incubation with 30 nM of the indicated antibodies. 20’000 events are visible per plot, and plots were generated using FlowJo V10 software. **(D–F)** Quantification of different quadrants. **(D)** Percentage of parent for Q1 (upper left) for CD2^-^/CD69^+^, **(E)** percentage of parent for Q2 (upper right) for CD25^+^/CD69^+^ events. **(F)** Percentage of parent for Q3 (lower right) for CD25^-^/CD69^+^ events. Circles represent the individual measurements, error bars represent the SD of biological duplicates from PBMC pools from four healthy donors.

A fundamental aspect of a T-cell engager is the ability of the CD3-binding moiety to activate T cells upon binding and engagement with tumour cells. To this end, a commercially available kit was used to measure activation of the NFAT pathway after engagement of effector (TCR/CD3 cells) and tumour cells (A431). Potent T-cell activation was observed for the two variants carrying only NI0401 with EC_50_ of 0.59 and 0.63 nM for (αCD3ϵ)K + H and K(αCD3ϵ)+H, respectively (**Figures 4A, B**). For the TriTECMs, a 5- and 10-fold reduction in EC_50_ was observed compared to their counterparts lacking anti-IL-6R scFvs, with 2.51 and 5.60 nM for (αCD3ϵ)K(αIL-6R) + H(αIL-6R) and K(αCD3ϵ) + H(αIL-6R), respectively (**Figures 4A, B**). Nonetheless, significant T-cell activation was observed, meaning no complete loss-of-function occurred through the diminished CD3 binding on effector cells (**Supplementary Table 1**).

While immortalised cell lines can provide an estimate of *in vitro* potency, human peripheral blood mononuclear cells (PBMCs) isolated from healthy donors are a more reliable source. Subsequently, PBMCs were incubated with 30 nM of the indicated antibodies for 48 h and the early and late stage T-cell activation markers CD69 and CD25, respectively, were measured by flow cytometry (**Figure 4C**). While the control antibodies and blank measurements all showed similar levels, a significant increase in both activation markers (upper right quadrant) was observed where the CD3-binding NI0401 scFv was present, as expected. Quantification of the different quadrants showed increased expression in CD25/CD69 and CD25 for all four NI0401-containing molecules, with a significant reduction for (αCD3ϵ)K(αIL-6R) + H(αIL-6R) compared to (αCD3ϵ)K + H, as previously observed (**Figures 4E, F**). Not many CD69^+^/CD25^-^ events were observed compared to the controls, however this could be due to the gradual decrease of CD69 with longer incubation times, as known from literature (78, 79) (**Figure 4D**).

In summary, T-cell activation was observed for all molecules with the CD3-binding NI0401 scFv, with the (αCD3ϵ)K(αIL-6R) + H(αIL-6R) showing the most attenuated T-cell activation, which might aid in the search for T-cell engagers with increased safety profiles.

### Attenuated cytokine release and cytotoxicity with TriTECMs

**Figure 5:**
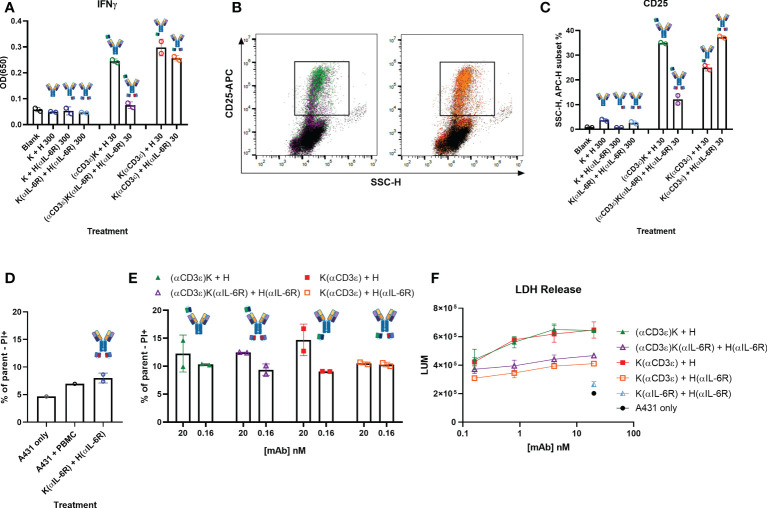
Cytokine release and T-cell-mediated cytotoxicity. **(A)** IFNγ secretion of PBMCs stimulated with the indicated construct and amount (in nM) for 72 h. **(B)** Dot plots showing side scatter (SSCs-H, x-axis) and CD25-APC (y-axis) of PBMCs after 72 h incubation. The colours represent the different antibodies as quantified in **(C)** C Quantification of the SSC-H, APC subset indicated in B as percentage of all events. **(D, E)** Dead cell staining by propidium iodide (PI) staining after 24 h co-incubation of A431 cells and PBMCs in an effector-to-target ratio of 10:1. The control antibody K(αIL-6R) + H(αIL-6R) was tested at 20 nM. **(F)** T-cell-mediated cytotoxicity of A431 cells after 48 h incubation with PBMCs and the indicated amounts of antibodies (10:1 effector-to-target ratio). A431 only represents the basal killing level. Circles represent the individual measurements, error bars represent the SD of biological duplicates from PBMC pools from four healthy donors.

One of the large drawbacks of T-cell engagers is the increase in pro-inflammatory cytokines such as TNFα and IFNγ upon T-cell activation, leading to the hyperactivation of macrophages. A hyperactivation of macrophages leads to the secretion of high levels of IL-6 and other cytokines, ultimately resulting in a cytokine storm. To investigate whether the addition of the anti-IL-6R scFv module resulted in decreased pro-inflammatory cytokine release, PBMCs were stimulated with 30 nM of the respective antibodies and stimulated over 72 h. Thereafter, the release of pro-inflammatory IFNγ was measured in the supernatant (**Figure 5A**), and the cells themselves were stained with an APC-conjugated anti-CD25 antibody to measure T-cell activation (**Figures 5B, C**). In both cases, significant increases in either IFNγ or CD25 were observed compared to the controls (K + H, K + H(αIL-6R), and K(αIL-6R) + H(αIL-6R)). In the case of IFNγ, a decrease was noted with the TriTECM (αCD3ϵ)K(αIL-6R) + H(αIL-6R) compared to (αCD3ϵ)K + H, hinting at both an attenuated activation and a decrease in cytokine release due to the anti-IL-6R scFv (**Figure 5A**). This effect was also noted for K(αCD3ϵ) + H(αIL-6R) compared to K(αCD3ϵ) + H but to a smaller extent, presumably due to monovalent IL-6R binding (**Figure 5A**). CD25 staining revealed significant T-cell activation with the TriTECM molecules compared to their controls (**Figure 5C**).

To ensure the attenuation of CD3-binding still led to decent T-cell-mediated cytotoxicity, cell killing of tumour cells by co-incubation with PBMCs was investigated. Dose-dependent cytotoxicity was observed through PI staining after 24 h co-incubation (**Figures 5D, E**; **Supplementary Figure 4**), with a slight reduction of activation for both TriTECM variants compared to their controls without Sar. Longer incubation times of 48 h revealed T-cell-mediated cytotoxicity as measured by lactate dehydrogenase (LDH) release (**Figure 5F**; **Supplementary Figure 5**). While the tetraspecific molecules resulted in lowered cytotoxicity, these results, combined with previous T-cell activation studies (**Figure 4**) prove the attenuated nature of TriTECMs and their potential use as TCEs with a cytokine release and activity modulating function.

## Discussion

With the increasing demand and interest for the development of new therapeutics in the fight against cancer, T-cell engagers and multispecific antibodies have been set in the spotlight as they are able to trigger and combine multiple mechanisms of action. For TCEs in particular, their safety profiles are often hindered by hyperstimulation of immune cells resulting in cytokine storms or cytokine release syndrome.

Within the scope of this work, proof-of-concept TriTECM molecules were designed consisting of a CD3-binding moiety derived from foralumab (71), an IL-6R binding module derived from sarilumab, and a bispecific two-in-one antibody binding both EGFR and PD-L1 (65). Production and purification of the antibodies as well as their respective controls using the KiH technology resulted in reasonable yields of pure product, considering the complexity of the molecules (80). High-affinity binding to both tumour targets, EGFR and PD-L1, as well as to IL-6R was observed *via* biolayer interferometry. On-cell affinities revealed high affinities to tumour cells but were decreased on both T cells and macrophages/monocytes, which could lead to improved tumour-penetration and accessibility into the tumour microenvironment without being captured before reaching the tumour site. Furthermore, the tetravalent TriTECMs were able to bind all antigens simultaneously. They retained the mechanism of action of the single antibody fragments when combined, including EGF- and IL-6 mediated signalling inhibition, and PD-L1/PD-1 axis blockade as determined by competition assays.

The design of the two reported TriTECMs vary mainly in their valency towards IL-6R, with (αCD3ϵ)K(αIL-6R) + H(αIL-6R) binding bivalently and K(αCD3ϵ) + H(αIL-6R) only monovalently. The effect of monovalent IL-6R binding was primarily seen in the competition assay *via* BLI and inhibition of IL-6-mediated signalling when compared to either sarilumab itself or (αCD3ϵ)K(αIL-6R) + H(αIL-6R), which resulted in much stronger competition as well as inhibition. Additionally, the positioning of the anti-CD3 scFv was considered, either being placed in close proximity to the TAA-engaging Fab arm by an N-terminal VH fusion [for (αCD3ϵ)K(αIL-6R) + H(αIL-6R)], or a C-terminal fusion to the CH3 domain of the Fc region [for K(αCD3ϵ) + H(αIL-6R)]. While previous results have identified higher potencies when the CD3-binder and the TAA-binder are in close proximity (75, 81), no significant differences were noted in the T-cell activation bioassay performed in this study. An explanation for this could be the different conformations of the molecule due to the fused scFvs, possibly bridging the molecule in closer proximity and thus having similar effects for both architectures.

An attenuated T-cell engager was developed which nonetheless resulted in T-cell activation and T-cell-mediated cytotoxicity as seen both on immortalized cell lines and human-derived PBMCs. The decreased affinity to CD3 on cells might also prevent the over-stimulation of immune cells, as is the case for next-generation TCEs which are being developed with lower affinities to avoid cytotoxicity (81). Secretion of T cell-released IFNγ was found to be significantly reduced after incubation with (αCD3ϵ)K(αIL-6R)+H(αIL-6R) compared to (αCD3ϵ)K+H, noting the potential of such a molecule to mitigate cytokine storm events when combined directly with IL-6R inhibition. Further understanding of the modulation *via* IL-6R remains to be investigated. The functionality of this type of molecule with such a large number of modules having different modes of action requires extensive animal studies which were out-of-scope for this proof-of-concept study.

While combination therapies with anti-IL-6R agents (tocilizumab, sarilumab) have been investigated and approved for therapeutics-induced cytokine release syndrome (31, 33, 40), simultaneous administration by being fused to the mAb itself might prove beneficial. Hypothetical clinical findings after CAR T-cell therapy report dramatic increases in cytokine levels 3 days after infusion, with tocilizumab having to be administered at day 6 or 7 (31, 82, 83). By using a full-length IgG1 backbone, the serum half-life of such a molecule would be approximately 21 days (84, 86), thus being in frame with the current management approaches or interventions required when grade 2 CRS is reached. Furthermore, the direct fusion of an anti-IL-6R that blocks further downstream signalling by inhibiting the JAK/STAT pathway responsible for the promotion of tumour growth and metastasis could provide beneficial anti-tumour properties. Such a synergistic effect may be promoted by direct fusion to tumour-targeting antibodies rather than co-administration, thereby enhancing the accumulation of an IL-6R blocking entity in the tumour microenvironment.

**Figure 6:**
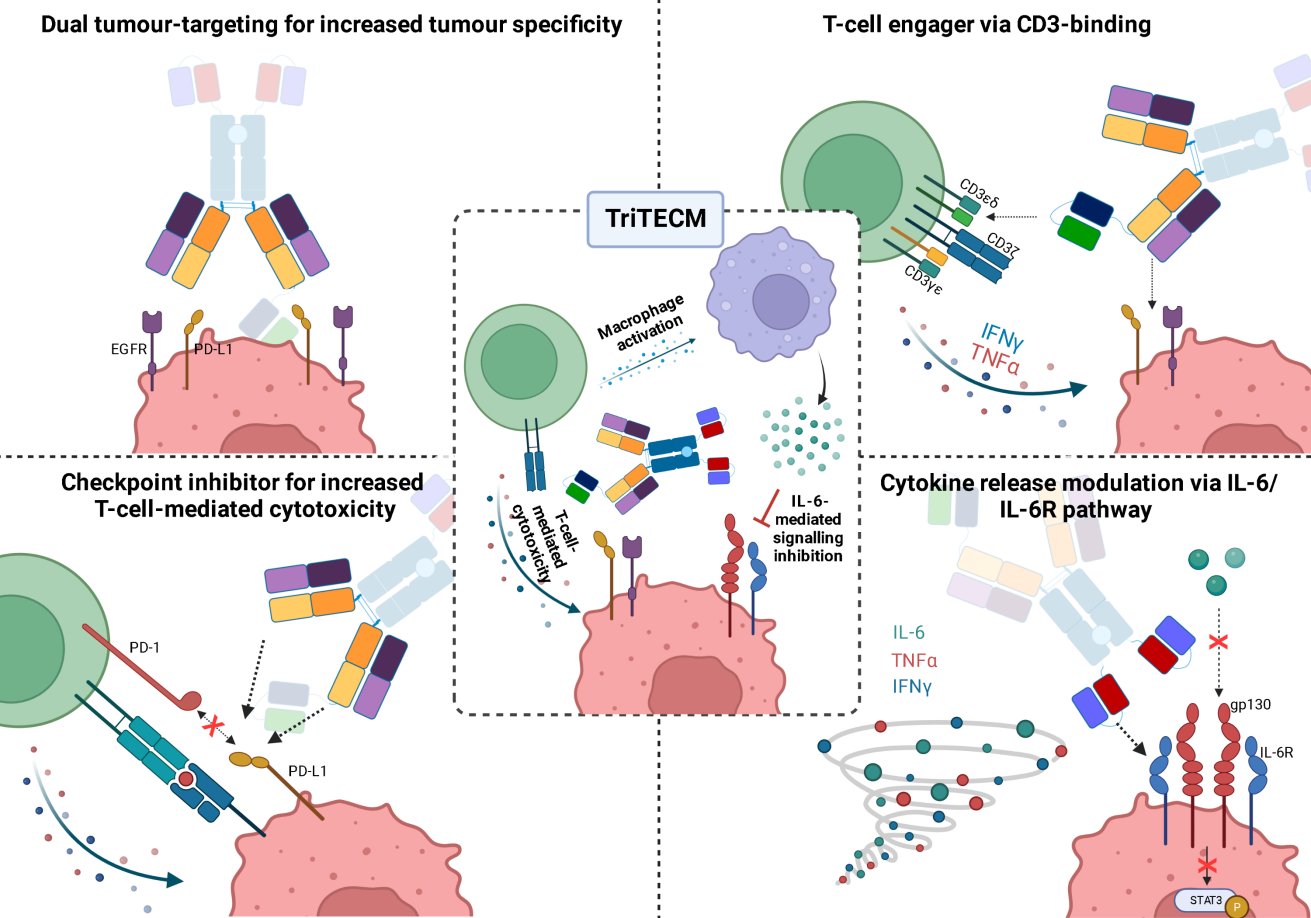
Schematic representation of the four functionalities of tetrafunctional TriTECM molecules, as exemplified by (αCD3ϵ)K(αIL-6R)+H(αIL-6R).

In summary, the tetrafunctional TriTECMs could consist of: i) dual tumour-targeting for increased tumour specificity by targeting EGFR and PD-L1, ii) engagement of T cells *via* CD3ϵ binding and bridging T cells in close proximity to tumour cells for tumour cell lysis, iii) increased T-cell-mediated cytotoxicity by blocking the PD-L1/PD-1 axis (checkpoint inhibitors), and iv) modulating cytokine storms or CRS by inhibiting the IL-6/IL-6R pathway by an anti-IL-6R scFv (**Figure 6**). Altogether, the advantages of TriTECMs may pave the way to finding novel biologicals for cancer therapies by harnessing the power of T cells. To further test this concept, other CD3-binding antibody fragments could be tested, and more importantly, already-existing antibodies which were shown to be toxic due to CRS or other side-effects might be able to be recycled in such a format due to the attenuation and modulation of both T-cell activation and IL-6-mediated signalling. Due to the attenuation of the CD3-binding moiety, bivalent binding to CD3 could also be investigated. In order to avoid unwanted on-target off-tumour effects *via* IL-6R binding to other cell types, further antibody engineering may be required to fine-tune the affinity of the IL-6R binding moiety. Alternative strategies might also include pH-responsive binders to only allow binding of the mAb to IL-6R within the acidified tumour microenvironment, or alternatively masking this binding unit with an anti-idiotypic binder connected *via* a cleavable linker. Ultimately, highly complex models are required to elucidate the efficacy of TriTECMs in modulating CRS *in vivo*. Additionally, pentafunctional molecules could be envisioned by using an Fc that induces effector functions and compare the effects of both variants on cytokine release and T-cell-mediated cytotoxicity.

## Data availability statement

The original contributions presented in the study are included in the article/**Supplementary Material**. Further inquiries can be directed to the corresponding author.

## Author contributions

SC and HK conceived and designed experiments. SC, JG and JH performed experiments. SC, BH and HK analysed the data. JG, BH and HK have scientific advice and guidance. SC wrote the manuscript. All authors contributed to the article and approved the submitted version.

## Acknowledgments

The authors would like to thank Janine Becker and Michael Ulitzka for their assistance in cell maintenance and antibody purification. Furthermore, we would like to thank the department of GPRD at Ferring Pharmaceuticals for funding. The funders played no role in study design, data collection/analysis, nor decision to publish. We acknowledge support by the Deutsche Forschungsgemeinschaft (DFG – German Research Foundation) and the Open Access Publishing Fund of the Technical University of Darmstadt.

## Conflict of interest

Authors SCC and JG were employed by company Ferring Pharmaceuticals. Author BH was employed by company Aerium Therapeutics. Authors SCC, JH, and HK are inventors of a patent related to the parental two-in-one antibody (EP22159491.4).

## Publisher’s note

All claims expressed in this article are solely those of the authors and do not necessarily represent those of their affiliated organizations, or those of the publisher, the editors and the reviewers. Any product that may be evaluated in this article, or claim that may be made by its manufacturer, is not guaranteed or endorsed by the publisher.
