# Supplementary material for: TriTECM: A tetrafunctional T-cell engaging antibody with built-in risk mitigation of cytokine release syndrome
Source: Front Immunol. 2022 Nov 10;13:1051875. doi: 10.3389/fimmu.2022.1051875 (PMC9687101; doi:10.3389/fimmu.2022.1051875)
Supplement: Supplementary file 1 [file DataSheet_1.pdf]

Supplementary Material

TriTECM: A Tetrafunctional T-Cell Engaging Antibody with Built-In Risk Mitigation of Cytokine Release Syndrome

Stefania C. Carrara[1,2], Julia Harwardt[1], Julius Grzeschik[3], Björn Hock[4], Harald Kolmar[1,5*]

1 Supplementary Figures and Tables

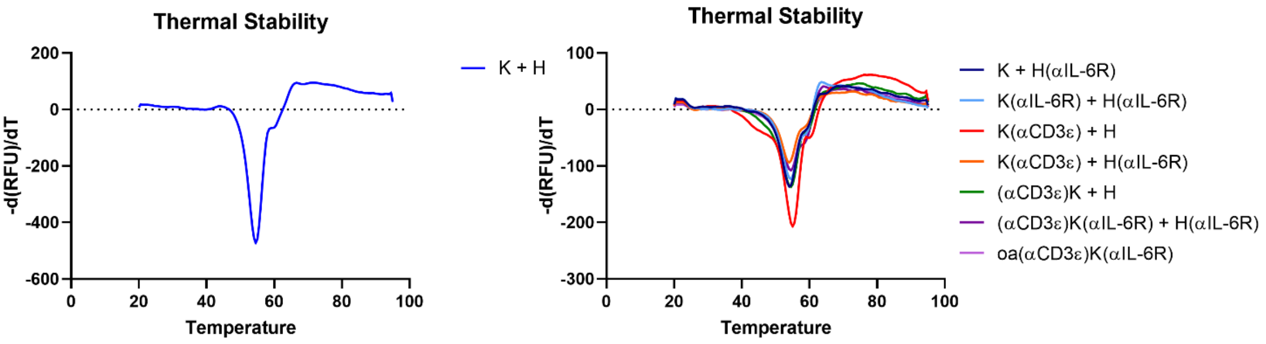

Supplementary Figure 1: Melt peaks after thermal shift assay by SYPRO Orange. RFU – relative fluorescence units. The derivative of RFU is plotted against temperature in a range from 20 – 95 °C. The dotted line represents the threshold set to determine the melt temperature.

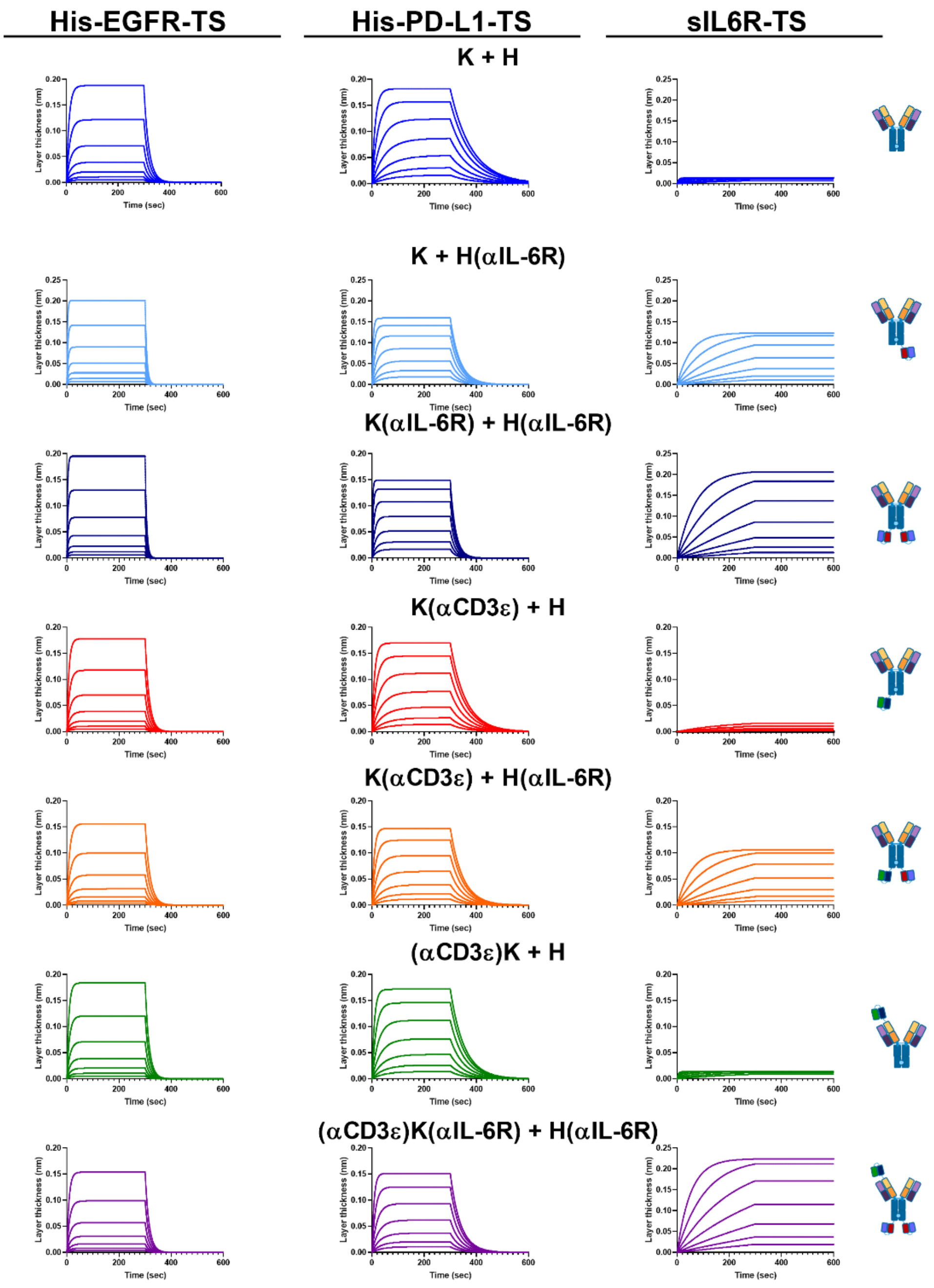

Supplementary Figure 2: Affinity determination by biolayer interferometry. The binding curves for His-EGFR-TS (left), His-PD-L1-TS (middle) or soluble IL6R-TS (right) are displayed for all antibodies. The colour-coding represents the different variants. For His-EGFR-TS and His-PD-L1-TS, a concentration range of 7.8 – 500 nM was measured, while for sIL6R-TS a range from 3.125 – 200 nM was measured.

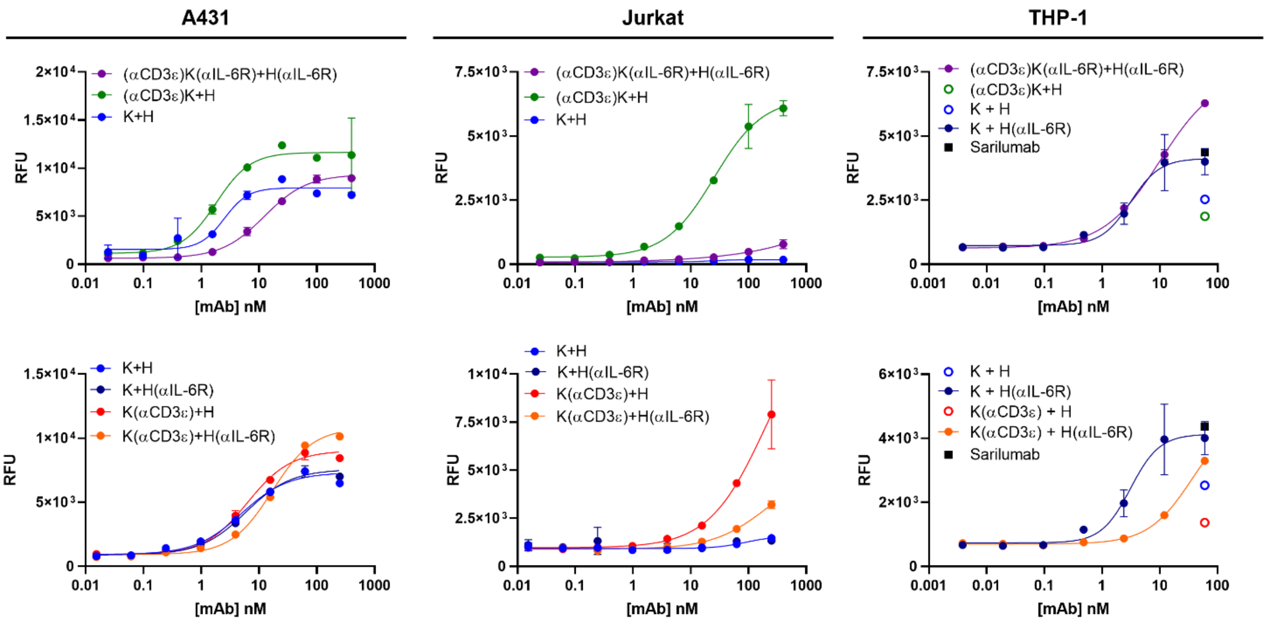

Supplementary Figure 3: On-cell titrations. The antibodies were tested for either CD3-binding (Jurkat), IL6R-binding (THP-1) or EGFR/PD-L1-binding (A431). The top panel show the variants with an N-terminal NI0401 scFv fusion, whereas the bottom panel shows the C-terminal NI0401 scFv variants with the respective controls. The mean fluorescence was determined and plotted using GraphPad prism. A non-linear regression was determined to calculate the on-cell affinities. RFU – relative fluorescence units.

Supplementary Table 1: On-cell affinities on A431 ($EGFR^{+++}$/$PD\text{-}L1^{+}$), Jurkat ($CD3^{+}$) and THP-1 ($IL6R^{+}$/$PD\text{-}L1^{+}$).

Variant	On-cell affinity (nM)		
	**A431**	**Jurkat**	**THP-1**
**K + H**	2.50	-	-
**K + H(αIL-6R)**	4.30	-	3.10
**K(αCD3ε) + H**	4.50	195.90	-
**K(αCD3ε) + H(αIL-6R)**	21.24	213.80	36.66
**(αCD3ε)K + H**	1.82	25.38	-
**(αCD3ε)K(αIL-6R) + H(αIL-6R)**	12.34	n.d.	10.08

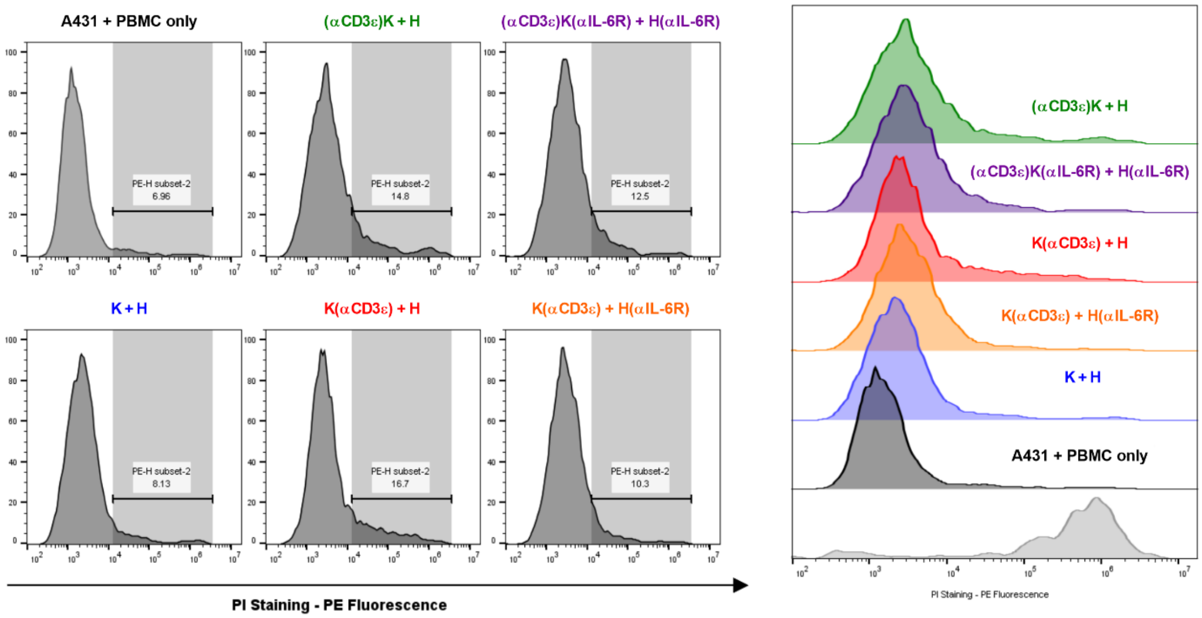

Supplementary Figure 4: Histograms and half-offset overlays for dead cell staining with propidium iodide (PI) after co-culture of A431 and PBMCs for 24 h to measure T-cell-mediated cytotoxicity. Histograms and overlays were generated using FlowJo V10 software.

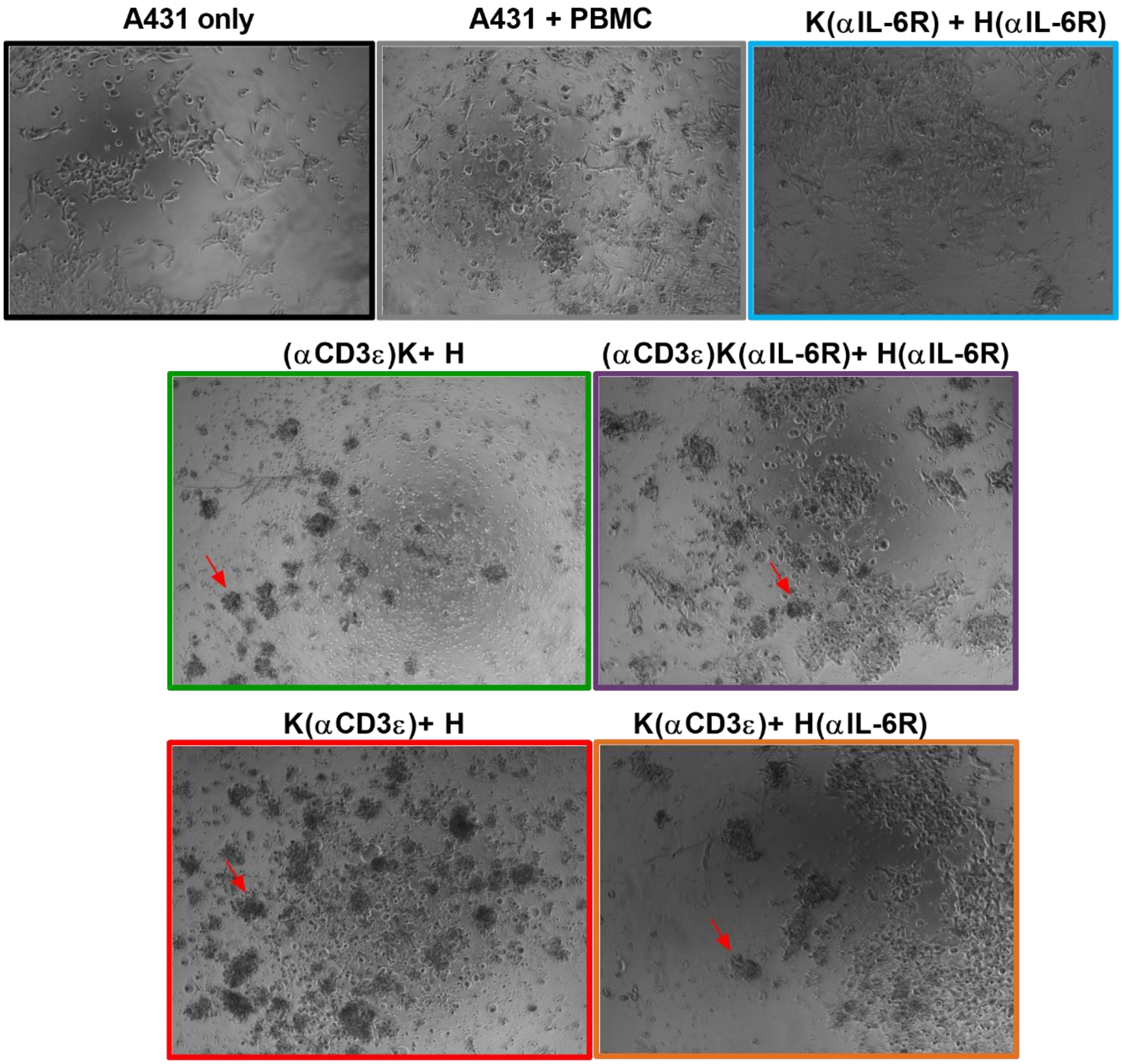

Supplementary Figure 5: Bright field images of A431 and PBMC co-culture in combination with 20 nM of the indicated antibodies after 48 h incubation. Black dead target cell clusters are exemplified by red arrows in each of the relevant images.
